# Understanding cyberbullying dynamics: risk factors and behavioral responses among nursing college students

**DOI:** 10.1186/s12912-025-03855-7

**Published:** 2025-10-06

**Authors:** Samira El Sayed El Mezayen, Aziza Ibrahim Abd El Kader Mohamed, Ayman Muhammad Kamel Senosy, Sahar Elsayed Gaber Behilak, Hend Reda Ali Elkest, Eslam Mohamed Ahmed Gaho

**Affiliations:** 1 Community Health Nursing, Faculty of Nursing, Tanta University, Tanta, Egypt; 2 Medical Surgical Nursing, Department of Nursing, Al Ghad College for Applied Medical Sciences, Jeddah, Saudi Arabia; 3 Medical Surgical Nursing at Faculty of Nursing, Cairo University, Cairo, Egypt; 4 Medical Surgical Nursing, Faculty of Nursing, Ain Shams University, Cairo, Egypt; 5 Faculty of Nursing, Galala University, Suez, Egypt; 6 Department of Nursing, College of Applied Medical Sciences, University of Jeddah, Jeddah, Saudi Arabia; 7 Psychiatric and Mental Health Nursing, Faculty of Nursing, Mansoura University, Mansoura, Egypt; 8 Department of Community Nursing, Faculty of Nursing, Tanta University, Tanta, Egypt; 9 Psychiatric and Mental Health Nursing, Faculty of Nursing, Tanta University, Tanta, Egypt

**Keywords:** Cyberbullying, Risk factors, Aggression, Attitude

## Abstract

**Background:**

Cyberbullying represents an important concern in the modern digital scenario, which includes harassment through electronic agents, which affects the recurrent aggression and humiliation of the selected individuals. Its widespread phenomenon among young adults, especially people who are registered with institutions for higher education, emphasizes the imperative for the intensive understanding of its underlying mechanisms.

**Aim:**

The study aimed to assess the understanding of cyberbullying dynamics and risk factors and examine behavioral reactions between nursing students in Egypt.

**Methods:**

A descriptive and analytical cross-sectional design was done. A cluster of 400 Egyptian nursing students from the Faculty of Nursing at Tanta University was involved in the random trial study. Data was collected from mid-February 2025 to mid-April 2025.

**Tools:**

Three tools, such as the cyberbullying dynamics and risk factor knowledge questionnaire, the cyber-aggression scale, and the cyberbullying attitude scale.

**Result:**

The study found that there was a highly statistically significant relation (*P* < 0.05) between cyber-aggression behavior and all items of students’ characteristics except age of studied students’ while there is a statistically significant relation (*P* < 0.05) between cyberbullying knowledge and age of studied students’ only. The findings suggested that cyberbullying dynamics and risk factors (χ² = 27,506, *p* < 0.001) and cyber activity behavior, as well as cyberbullying approaches and trust and cyber-aggression behavior (χ² = 91,460, *p* < 0.001), were learned.

**Conclusion:**

This study highlights the significant relationship between nursing students and cyberbullying, their approach, and participation in cyber-invasive behavior. Conclusions emphasize the need for targeted educational intervention and training programs to increase knowledge, promote a negative attitude towards online bullying, and equip future nurses with skills to effectively address the problem.

**Clinical trial number:**

Not applicable.

## Introduction

Cyberbullying is an emerging phenomenon that has garnered increasing attention in academic and social discourse due to its profound impact on individuals, particularly students [1]. This pervasive issue leverages social media and digital communication platforms to repeatedly and intentionally harass, injure, embarrass, humiliate, or frighten another person. Specifically, cyberbullying is defined as the repeated and intentional use of digital communication platforms to harass, injure, embarrass, humiliate, or frighten another person [2, 3].

Cyberbullying encompasses a range of harmful behaviors facilitated through digital media. This includes sending, posting, or sharing negative, harmful, or false content about an individual. Digital media covers both devices, such as smartphones and laptops, and applications like Snapchat and Instagram, which enable the creation, consumption, and exchange of information in digital format. These platforms play a crucial role in peer interactions, games, and self-expression among young people [4].

Within this context, two crucial related concepts are cyberbullying attitudes and cyber-aggression. Cyberbullying attitudes refer to an individual’s perceptions regarding the acceptability, harmlessness, or potential social power derived from engaging in cyberbullying behaviors [5]. These attitudes can be influenced by factors such as anonymity, a perceived lack of consequences, and emotional detachment from the victim’s response. Consequently, a permissive attitude can fuel cyber-aggression, defined as the intentional use of electronic means to harm, harass, or humiliate others [6].

Globally, digital platform engagement is substantial. According to the Global Digital Report, the number of social media users worldwide reached 3.484 billion in 2019, marking a 9% increase over the previous year [7]. The Arab region demonstrates similarly high and growing engagement. For instance, in January 2020, the Middle East and North Africa (MENA) region saw continued growth in social media adoption, with users spending an average of over 3.5 h daily on social networks, indicating a deeply embedded digital culture [8, 9]. Specific countries like Saudi Arabia reported 25 million social media users in January 2020, an 8.7% increase from the previous year [9]. In Egypt, the digital landscape in 2020 included 48.5 million internet users, with 35 million actively engaging on Facebook [10, 11].

While global statistics highlight the issue, cyberbullying is also a significant concern in Arab countries. Studies indicate a growing prevalence among youth in the region. For example, a 2019 survey in Dubai, UAE, found that 14.8% of adolescents experienced cyberbullying, constituting 65.5% of all bullying incidents among that demographic [12]. Similarly, a study among female college students in Saudi Arabia revealed that 41.6% had encountered some form of cyberbullying at least once in their lifetime [13]. In Egypt, research among high school students in Zagazig reported a cyberbullying victimization prevalence of 38.3% [14]. These figures underscore the urgent need to address this problem within the unique cultural and social contexts of the Arab world.

Cyberbullying is a pressing issue among college students, with research indicating that many have experienced or witnessed such incidents during their academic journey [15]. Young people aged 15 to 24 are often targeted deliberately in cases of cyberbullying [16, 17]. The prevalence of cyberbullying among college students remains significant. In Canada, a third of 15-29-year-olds reported being victims of cyberbullying [18], while Pew Research Center data suggest that the US rate is almost twice as high [19].

On social media platforms, young adults found cyberbullying at various rates: 42% on Instagram, 37% on Facebook, 31% on Snapchat, and 9% on Twitter Understanding what drives cyberbullying is essential for stopping it and helping those affected. These contributing factors, or risk factors, fall into categories like social media habits, psychological vulnerabilities, and environmental influences. Knowing about these specific risks is vital for individuals, educators, and parents so they can spot, address, and ultimately reduce cyberbullying and its impact [20].

Research identifies several inherent social media dynamics that can encourage or facilitate cyberbullying behavior. These include anonymity/pseudonymity, where hiding one’s identity reduces inhibitions and increases aggressive conduct. The broad audience reach of these platforms allows content, positive or negative, to spread rapidly, amplifying the impact of bullying [21]. A significant factor is the lack of non-verbal cues in online interactions, as the absence of face-to-face communication can lead to misinterpretation and dehumanization of the victim [22].

Furthermore, the persistence of content means material posted online is difficult to remove, causing prolonged suffering. The bystander effect is also prevalent, where a large online audience can diffuse responsibility, making individuals less likely to intervene. Finally, instant gratification and reactions from feedback loops, like likes, shares, and comments, can unfortunately reinforce bullying behavior [23]. Furthermore, peer pressure from online “colleagues” or groups can compel individuals to participate, and in some instances, simple boredom or a desire for entertainment can also drive cyberbullying behaviors [24, 25].

The dynamics of cyberbullying involve distinct roles within this cycle of violence. These typically include the aggressor (perpetrator), the victim, and bystanders (those who observe and sometimes share harmful content). Teachers and parents are also key figures, though they are sometimes the last to become aware of misuse [26]. Several studies have highlighted many long-term negative consequences associated with involvement in cyberbullying, both as a perpetrator and a victim [27]. These consequences can include adverse effects on educational performance, psychological and mental problems such as depression and anxiety, reduced life satisfaction and self-esteem, and self-conscious behavior [28, 29].

Moreover, a recent systematic review [30] underlined that students involved in cyberbullying are at greater risk of suicidal ideation and suicide attempts than those not involved. Significantly, substance abuse, such as alcohol, tobacco, and cannabis use, is also among the main negative behavioral consequences associated with young people involved as cyberbullies [28]. Nursing students are particularly concerned about cyberbullying since it affects their social, mental, and academic health. A significant portion of nursing students have been victims of cyberbullying, which can have detrimental effects like loneliness, anxiety, and sadness.

According to the study’s findings [31], most nursing students had academic, psychological, and social issues because of cyberbullying. Thus, it’s critical to educate students and their parents about responsible internet use, cyberbullying, and how it affects both academic performance and health. About 30.1% of the kids did not engage in any form of cyberbullying, whereas 8.6% were classified as perpetrators of cyberbullying alone, 20.4% as victims, and 40.9% as both perpetrators and victims. Among the nursing students under study, verbal/written victimization and visual/sexual perpetration were the most common types of cyberbullying.

## Theoretical framework

This study is framed primarily within Social Cognitive Theory (SCT), proposed by Albert Bandura. SCT posits that human behavior is acquired through a continuous interaction among cognitive, behavioral, and environmental influences. In the context of cyberbullying, SCT suggests that individuals’ knowledge of cyberbullying dynamics and risk factors (cognitive factors) can shape their attitudes (beliefs and values) towards such behaviors. Furthermore, these cognitive and affective components, influenced by observing others and self-regulatory mechanisms (e.g., self-efficacy for resisting negative online pressures), are instrumental in predicting an individual’s behavioral responses, including engagement in or avoidance of cyber-aggression. This theoretical lens allows us to explore how awareness and perceptions of cyberbullying can lead to more responsible online conduct among nursing students [32].

### Study hypotheses

Based on the theoretical framework and existing literature, the following hypotheses were formulated:

#### H1

Certain demographic characteristics are significantly associated with nursing students’ knowledge of cyberbullying dynamics and risk factors, cyber-aggression behaviors, and cyberbullying attitude.

#### H2

There is a significant relationship between nursing students’ knowledge of cyberbullying dynamics and risk factors and their cyber-aggression behaviors.

#### H3

There is a significant relationship between nursing students’ attitudes towards cyberbullying and their cyber-aggression behaviors.

### Significant of the study

Nursing is a demanding profession, and cyberbullying can significantly affect mental health, academic performance, and the general well-being of nursing students. This study can identify specific risk factors and behavioral responses, allowing interventions directed to protect these future health service providers from the harmful effects of cyberbullying. Healthy nursing students are more likely to become effective and compassionate nurses [33]. Given Egypt’s rapidly expanding healthcare sector and its critical need for a robust, resilient nursing workforce, understanding and mitigating these threats is paramount. This is further amplified across the Arab region, where similar pressures on healthcare systems necessitate strong mental health support for professionals in training [34]. A nurse whose mental well-being is compromised due to cyberbullying may be less attentive or prone to errors [35].

In the context of Egypt’s public health challenges and the region’s focus on healthcare development, ensuring nurses are psychologically well-prepared for their roles directly impacts national health outcomes. The findings are particularly salient for Egypt, a country undergoing significant healthcare reform, including initiatives to boost the quality and capacity of its nursing staff. By addressing cyberbullying, this research directly supports national health goals related to workforce development and patient safety within the Egyptian context.

It can create a hostile and unsupportive learning environment, hinder academic progress and foster feelings of isolation. By addressing cyberbullying, the study contributes to fostering a more positive, inclusive, and supportive educational setting for nursing students, which is essential for their holistic development [33]. This is particularly pertinent in educational systems like Egypt’s, where traditional hierarchical structures might complicate student reporting mechanisms, making a proactive approach to fostering supportive environments even more vital [36].

The findings of this research can provide evidence-based insights for nursing college administrators and educators to formulate and implement effective policies and guidelines regarding cyberbullying. This includes developing clear codes of conduct, establishing reporting procedures, and providing support services for victims and even perpetrators [37].

Crucially, this study will contribute original, localized data to the understanding of cyberbullying among nursing students in Egypt, a context often underrepresented in global research. The findings will provide specific, actionable recommendations tailored to the cultural nuances and educational systems prevalent not only in Egypt but also in similar Arab countries, thereby enriching regional literature and informing context-specific policy development.

Study objectives

1. To measure university nursing students’ understanding of cyberbullying dynamics and associated risk factors.
2. To measure the attitudes towards cyberbullying among university nursing students.
3. To assess the prevalence rate of self-reported cyber-aggression behaviors among university nursing students.
4. To examine the statistical correlation between university nursing students’ understanding of cyberbullying dynamics and risk factors and their engagement in cyber-aggression behaviors.
5. To examine the statistical correlation between students’ attitudes towards cyberbullying and their engagement in cyber-aggression behaviors.

## Methods

### Research design

This study employed a descriptive and analytical cross-sectional design following STROBE guidelines. This approach was chosen to capture a snapshot of cyberbullying dynamics, associated risk factors, and reported behavioral responses among nursing college students at a single point in time. This design is appropriate for describing the prevalence of these phenomena and exploring associations between variables within the study population [38]. It is highly suitable as it allows for the efficient and practical assessment of many students across different academic years without disrupting their demanding educational schedules. It provides crucial baseline data on cyberbullying prevalence within this high-risk group [39].

### Setting

The study was held in the faculty of nursing Tanta University, which is affiliated with the Ministry of Higher Education and Scientific Research. Tanta University is one of Egypt’s prominent public universities, located in Tanta City, within the Gharbia Governorate of the Nile Delta region. Established in 1962, it serves a large student body across various disciplines, contributing significantly to education and research in the central Delta region.

### Sampling

The required sample size was calculated using a standard formula for estimating a population proportion, assuming a 5% margin of error, a 95% confidence level, and a 50% response distribution [36]. Based on a total undergraduate nursing student population of 4006 for the academic year 2024–2025, the minimum calculated sample size was determined to be 351 participants. To further increase the statistical validity and strength of the study’s findings, this number was purposively increased to 400 participants.

A multi-stage sampling technique was employed to select the study participants [40]. The total student population of 4006 was distributed across four academic years: first year (881 students), second year (982 students), third year (1113 students), and fourth year (1030 students).

Stage 1: Stratified Cluster Sampling of Academic Years. Initially, each academic year was treated as a stratum. To ensure proportionate representation of students from each academic year and to maintain the heterogeneity of the overall student body within the initial selection, approximately 10% of the students from each academic year were systematically selected as initial clusters. This approach allowed for a manageable and representative selection of clusters while preserving the proportional distribution across the academic levels [41]. This resulted in the selection of clusters corresponding to first year: 88 students (from 881); second year: 98 students (from 982); third year: 111 students (from 1113) and fourth year: 103 students (from 1030). This yielded an initial selection of approximately 400 students across these clusters, effectively meeting and slightly exceeding the calculated minimum sample size of 351 while ensuring adequate representation from all academic stages.

Stage 2: simple random sampling within clusters. From the clusters selected in the first stage, simple random sampling was then used to select the final 400 participants. This was achieved by obtaining a complete list of student identification numbers from each selected cluster (academic year). To ensure pure randomness and an equal chance of selection for every student within each chosen cluster, these identification numbers were entered into a digital spreadsheet. A reliable online random number generator was then employed to generate unique random numbers corresponding to the number of participants required from that specific cluster. The student identification numbers matching these generated random numbers were then selected for inclusion in the study. This process was repeated for each academic year’s cluster until the target of 400 participants was reached.

### Tools of data collection

The study employed three data collection tools to comprehensively assess cyberbullying dynamics, risk factors, aggression, and attitudes among nursing college students.

#### Tool I: cyberbullying dynamics and risk factor knowledge questionnaire

This questionnaire was developed by researchers based on a comprehensive review of the current literature on cyberbullying [42, 43]. It is designed to assess nursing students’ knowledge regarding various aspects of cyberbullying and their awareness of associated risk factors. The questionnaire consists of three main parts:

**Part 1: Socio-demographic characteristics form** This section gathers essential descriptive information about the students, including age, gender, academic year, and residence.

**Part 2: Cyberbullying dynamics questionnaire.** This part comprises 32 items divided into several sub-sections to assess knowledge about the core aspects of cyberbullying. Specifically, it includes items on the definition of cyberbullying (2 questions: Q1, Q2), vulnerable groups (4 questions: Q3-Q6), general cyberbullying dynamics (6 questions: Q7-Q12), warning signs (5 questions: Q13-Q17), negative impacts (6 questions: Q18-Q23), and prevention strategies (9 questions: Q24-Q32).

**Part 3: Cyberbullying risk factors questionnaire.** This section consists of nine questions aimed at evaluating students’ awareness of different cyberbullying risk factors, informed by recent literature [41, 42]. These questions are categorized into three major areas: personal risk factors (4 questions: Q33-Q36), peer/colleague risk factors (2 questions: Q37-Q38), and environmental risk factors (3 questions: Q39-Q41).

##### Scoring system (Tool I)

Parts 2 and 3 of the Cyberbullying Dynamics and Risk Factor Knowledge Questionnaire are scored based on correct responses. Each correct answer is allocated one point, resulting in a total score ranging from 0 to 41. These raw scores are then converted into percentages and classified into three levels of knowledge.

- Limited knowledge: Below 50% of the total score.
- Satisfactory knowledge: Between 50% and 75% of the total score.
- Deep knowledge: Above 75% of the total score [44].

#### Tool II: Cyber-aggression scale (CYBAGS)

This self-report instrument, developed by [45], is designed to measure the perpetration of cyberbullying behaviors among adolescents, typically assessing behaviors within the last 12 months. It consists of 18 items that assess both direct (e.g., sending hurtful messages) and indirect (e.g., spreading rumors online) forms of cyber-aggression.

##### Scoring system (Tool II)

The Cyber-Aggression Scale uses a 5-point Likert-type scale where respondents indicate the frequency of each behavior, ranging from 1 = Never to 5 = More than 10 times. Total scores range from 18 to 90, derived by summing responses across all 18 items. Higher scores indicate a higher frequency of self-reported cyber-aggressive behaviors [23]. The overall score is categorized as follows.

- 18–24: Low cyber-aggressive behavior.
- 25–30: Moderate cyber-aggressive behavior.
- 31–90: Highly cyber-aggressive behavior.

#### Tool III: cyberbullying attitude scale (CAS)

Developed by [46], this scale aims to evaluate self-reported attitudes towards cyberbullying situations. The measure comprises nine items distributed across two factors: (1) negative cyberbullying attitudes and (2) general cyberbullying characteristics.

##### Scoring system (Tool III)

The Cyberbullying Attitude Scale uses a 5-point Likert scale where respondents indicate their level of agreement with statements, ranging from 1 = Strongly Agree to 5 = Strongly Disagree. Total scores are calculated by summing the responses to all items and range from 9 to 45. The interpretation of scores is as follows.

- Higher scores (25–45): Indicate more positive attitudes towards cyberbullying (suggesting a greater likelihood of engaging in or condoning cyberbullying behaviors).
- Lower scores (9–24): Indicate negative attitudes towards cyberbullying (suggesting a lower likelihood of getting involved in such behaviors).

### Tools validity and reliability

Prior to their use in the main study, all scales underwent a rigorous translation and back-translation process by independent bilingual experts to ensure linguistic and conceptual equivalence. Subsequently, all three tools were piloted on a small sample of 40 nursing students from Tanta University (who were not part of the main study sample) to assess clarity, comprehensibility, and cultural appropriateness of the items, as well as to establish preliminary psychometric properties. Revisions were made based on pilot study feedback.

#### Tool I: cyberbullying dynamics and risk factor knowledge questionnaire

The preliminary psychometric properties of this researcher-developed questionnaire were established through the pilot study. Internal consistency, as measured by Cronbach’s Alpha, was found to be 0.75 for the overall scale, indicating acceptable reliability for a newly developed instrument in this context. Content validity was ensured through expert review by five academic experts in nursing and psychology.

#### Tool II: Cyber-aggression scale (CYBAGS)

Cronbach’s alpha for the original global scale was acceptable (0.88) [45]. In the present study, following its translation and pilot testing, the scale demonstrated good internal consistency with a Cronbach’s Alpha of 0.78.

#### Tool III: cyberbullying attitude scale (CAS)

The original scale reported internal consistency rates ranging from 0.71 to 0.94 for negative cyberbullying attitudes and from 0.62 to 0.77 for general cyberbullying characteristics [46]. In this study, following translation and pilot testing, the CAS demonstrated acceptable internal consistency with a Cronbach’s Alpha of 0.73 for the overall scale.

### Pilot study

A preliminary pilot study was carried out at the beginning of the research, involving 10% of the total participants (40 students). This pilot aimed to check if the study tools were clear, practical, thorough, unbiased, and sufficient, and to see how well they could be applied. It helped pinpoint any issues with the methods or tools and figure out how long it would take to complete them. Interestingly, no changes were needed after the pilot. To maintain data integrity, participants from the pilot study weren’t included in the main research. The researchers also carefully reviewed the questionnaires to ensure their accuracy and comprehensiveness.

### Statistical analysis

The collected data were analyzed utilizing the Statistical Package for the Social Sciences (SPSS) version (28). Descriptive statistics (frequencies, percentages, means, standard deviations) were used to summarize the socio-demographic characteristics of the participants and their levels of knowledge, attitudes, and cyber-aggression behaviors. Chi-square (χ2) tests were employed to examine the associations between the categorical variables, specifically the level of knowledge about cyberbullying dynamics and risk factors and cyber-aggression behaviors, and the attitudes and beliefs towards cyberbullying and cyber-aggression behaviors. The significance level was set at *p* < 0.05.

### Data collection procedure

Data collection was conducted directly by the investigator, ensuring immediate oversight and the ability to address any student queries in real-time. Each academic level was visited over a two-week period, three times per week, with sessions strategically scheduled after students had completed their clinical rotations and were meeting with their instructors at the faculty. This timing maximizes student availability and minimizes disruption to their demanding academic schedules.

During these sessions, which typically involved groups of 20–30 students in a designated quiet common area, students first received a comprehensive, standardized face-to-face verbal explanation about the study. This included its purpose, significance, and what their participation would entail, with a particular emphasis on their right to voluntary participation and the confidentiality of their responses.

Students were given the opportunity to ask questions, which primarily revolved around clarifying specific questionnaire items or confirming the study’s scope; care was taken to provide non-leading responses. This non-directive approach was critical in preventing any influence on participant responses and fostering an atmosphere of trust conducive to honest self-reporting. The fieldwork for this study spanned approximately two months, commencing in mid-February 2025 and concluding by mid-April 2025. During this period, the investigator maintained a consistent presence to facilitate the process, address any emergent concerns, and ensure the integrity and completeness of the data collected.

### Result

**Table 1:** Frequency and percentage distribution of demographic characteristics (*n* = 400)

Students’ characteristics	No.	%
**Age (yrs**)		
18 - < 19 yrs.	47	11.75
19 - < 20 yrs.	64	16.0
20 - < 21yrs.	47	11.75
≥ 21 yrs.	242	60.5
Mean ± SD	20.69 ± 1.66	
**Gender**		
Male	85	21.25
Female	315	78.75
**Academic year**		
1st level	88	22
2nd level	98	24.5
3rd level	111	27.75
4th level	103	25.75
**Marital status**		
Single	358	89.5
Married	42	10.5
**Residence**		
Rural	312	78.0
Urban	88	22.0
**Family income**		
Enough and save	108	27.0
Enough	259	64.75
Not enough	33	8.25
**Have you heard about cyberbullying?**		
Yes	366	91.5
No	34	8.5
**Have you been subjected to cyberbullying?**		
Yes	38	9.5
No	362	90.5
**Have you ever cyberbullied someone?**		
Yes	21	5.25
No	379	94.75

Table 1 illustrates that 78.75% and 78% of the students were female and from rural area respectively. While 89.5% were single, 60.5% and 64.75% of them their age ≥ 21 years and have enough family income. Regarding the academic level, 28% & 25.5% of the students studied at third and fourth academic levels. In relation to cyberbullying 91.5%, 90.5% & 94.75% heard about cyberbullying and are not subjected to cyberbullying or cyberbullied someone.

**Table 2:** Chi-square analysis of associations between student demographic characteristics and cyberbullying dynamics and risk factors knowledge, Cyber-Aggression behaviors, and cyberbullying attitudes (*n* = 400)

Students’ characteristics	Cyberbullying dynamics and risk factors knowledge		Cyber-aggression behaviors		Cyberbullying attitude	
	χ 2	*P*- value	χ 2	*P*- value	χ 2	*P*- value
Age (year)	15.494	0.050*	19.89	0.061	4.026	0.285
Gender	0.830	0.660	4.703	0.000*	0.193	0.660
Academic year	9.340	0.314	16.56	0.035*	9.558	0.049*
Marital status	1.903	0.386	4.674	0.000*	0.240	0.624
Residence	2.222	0.329	4.742	0.000*	2.174	0.140
Family income	5.025	0.285	4.733	0.000*	1.425	0.491
Have you heard about cyberbullying?	2.871	0.238	4.683	0.000*	0.384	0.535
Have you been subjected to cyberbullying?	0.263	0.877	4.662	0.000*	0.064	0.800
Have you ever cyberbullied someone?	0.704	0.703	4.678	0.000*	1.405	0.236

*P-Value important at 0.05

Table 2 presents the chi-square correlation analysis examining the relationships between various demographic characteristics of the study participants and their levels of Cyberbullying Dynamics and Risk Factors Knowledge, Cyber-Aggression behaviors, and Cyberbullying Attitudes. The findings reveal several significant associations. It displays that there is a highly statistically significant relation (*P* < 0.05) between cyber-aggression behavior and all items of students’ characteristics except age of studied students’ while there is a statistically significant relation (*P* < 0.05) between cyberbullying knowledge and age of studied students’ only. Also, there is a statistically significant relation (*P* < 0.05) between cyberbullying attitudes and studied students’ academic year respectively.

**Table 3:** Cyberbullying dynamics and risk factor knowledge and frequency and percentage distribution of cyberbullying approach (*N* = 400)

Variables	No	(%)
**Cyberbullying dynamics and risk factors knowledge**		
Limited knowledge **(**< 50%)	40	10
Satisfactory knowledge (50% - >75%)	67	16.75
Profound knowledge ≥ 75%	293	73.25
**Cyberbullying attitudes**		
Negative attitude (9–24)	322	80.5
Positive attitude (25–45)	78	19.5

Table 3 shows that 73.25% of the students had profound knowledge regarding cyberbullying dynamics & risk factors. In relation to cyberbullying attitudes, 80.5% of the students had a negative attitude toward cyberbullying.

**Fig. 1:**
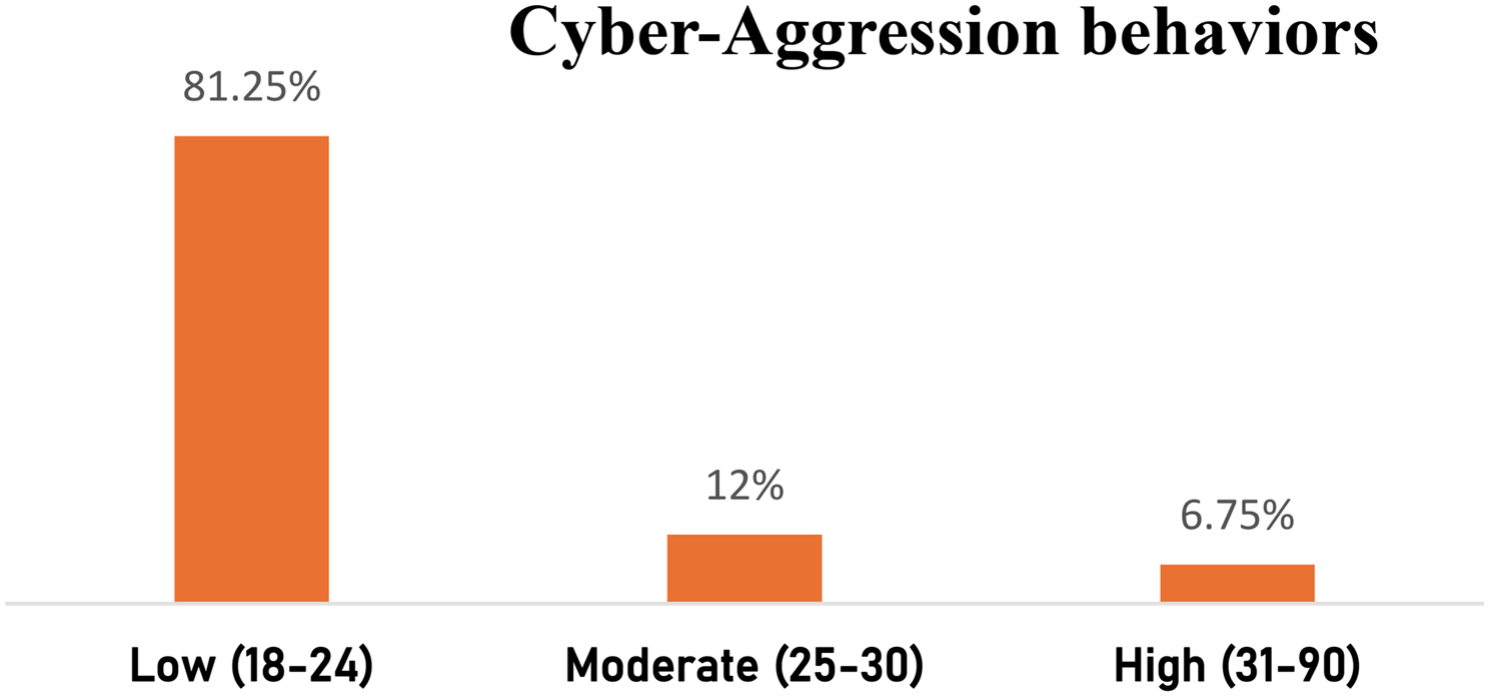
Percentage distribution of cyber-aggression behaviors among study samples (*n* = 400)

Figure 1 represents that 81.25% of the students studied had low cyber-aggression behaviors.

**Table 4:** Chi-square correlation among cyberbullying dynamics and risk factors knowledge, cyberbullying attitude and cyber-aggression behaviors among study sample (*n* = 400)

Variables	Cyber-aggression behaviors	
	χ 2	*P*- value
Cyberbullying Dynamics and Risk Factors Knowledge	27.506	0.000*
Cyberbullying attitudes	91.460	0.000*

* Important at P-Value ≤ 0.05

Table 4 represents statistically significant associations were found between knowledge of cyberbullying dynamics and risk factors (χ2 = 27.506, *p* < 0.001) and cyber-aggression behaviors, as well as between cyberbullying attitudes and beliefs and cyber-aggression behaviors (χ2 = 91.460, *p* < 0.001).

**Table 5:** Linear regression analysis predicting cyber-aggression behaviors (*n* = 400)

Variables	B	SE	β	t	*P*-value	95% confidence interval
(Constant)	25.10	2.50		10.04	< 0.001	(20.19, 30.01)
Cyberbullying Dynamics and Risk Factors Knowledge	−0.1360	0.0388	-0.18	-3.505	0.0065	(-0.212, -0.059)
Cyberbullying attitudes	−0.4757	0.0339	-0.45	-14.032	0.0000	(-0.542, -0.409)
R	0.476					
R2	0.2263					
Adjusted R-squared	0.222					

Table 5 presents the results of a linear regression analysis examining the factors that predict cyber-aggression behaviors. It displays statistically significant negative relationship between Cyberbullying Dynamics and Risk Factors Knowledge and cyber-aggression, with a B coefficient of -0.1360 (*p* = 0.0065), indicating that a greater understanding of these factors is associated with a decrease in aggressive behaviors. Even more impactful is the negative relationship with Cyberbullying attitudes, which has a B coefficient of -0.4757 (*p* < 0.001).

## Discussion

The dynamics of cyberbullying among nursing college students show notable behavioral and psychological effects, and risk variables include technology use patterns, views of the social environment, and personal vulnerabilities. Anxiety, despair, social disengagement, and possible academic disengagement are among the repercussions. Self-worth, encouraging peer interactions, and useful coping techniques are examples of protective factors. The study assessed the understanding of cyberbullying dynamics and risk factors and found out behavioral reactions between nursing students. And the study explored the significant relationship between nursing students such as cyberbullying, their approach and participation in cyber-invasive behavior.

Understanding cyberbullying in Egypt requires localizing the discussion to its cultural, religious, and educational contexts. Egyptian culture, being largely collectivistic, emphasizes group harmony and respect for authority, which can lead to underreporting of cyberbullying due to a desire to avoid confrontation or protect family honor. The religious context of Islam, with its strong emphasis on moral conduct and prohibitions against harmful speech, implicitly shapes students’ perceptions of cyberbullying as an unethical act, influencing their attitudes and behaviors online [23]. Finally, the educational environment within Egyptian nursing schools, characterized by its hierarchical structure and the demanding nature of the profession, may impact reporting behaviors and coping mechanisms, as students navigate pressures and internalize professional ethics that can conflict with experiences of cyberbullying [36].

In Egypt, Arafa found in his study that the cyberbullying victimization is highly prevalent amongst university students in Beni-Suef and female students are more vulnerable to exposure [36]. And [47] through a cross-sectional study published in The Egyptian Family Medicine Journal found a high prevalence of bullying among university medical students, with the verbal type being the most common. Another study, published in Mansoura Journal of Forensic Medicine and Clinical Toxicology, reported that most students (85.4%) had experienced bullying, with 31.1% admitting to bullying others.

This study suggests that three-quarters of the study sample were women and from rural areas. These results [48] were in line with the study, which reported that most of the study samples were young single women. This is responsible for the fact that in the past, the nursing profession depended on the female workforce compared to men, so women have a higher opportunity and are more ready to study than men. Also, a study conducted by [23] showed that two-thirds were women, and 56.1% lived in rural areas and 29.2% lived in urban areas. This discovery was according to [49], who said that 135 women of a total of 412 students (32.77%) participated in the study. Apart from this, about two-thirds of them are their age, 21 years.

In this study, the majority in relation to cyberbullying had heard of cyberbullying, and no one is subjected to cyberbullying or cyberbullies. It is accountable for people who do not interact with cyberbullies. Control online privacy settings, stay safe online, secure passwords, improve the university environment, and raise parental awareness and supervision to stop other students’ social support and cyberbullying so that students use it wisely at home.

This [23] comes with, who revealed that about three-quarters of the study subjects were not cyberbullied and they did not do cyberbullying. Whereas about three-quarters had not heard of cyberbullying. Similarly, 16% of the respondents accepted in a study conducted by [50] engage in two or more cyberbullying activities during their university education. This discovery is incompatible with past research by [51], who reported that 52% of 12- to 19-year-old children (*N* = 408) experienced online bullying, 20% of which were accountable with the Internet Chat Room for 20% of the events.

This study features a highly statistically significant relationship (*P* < 0.05) among all the items of students except cyber-curb behavior and studied students, as there are no stages of development that are interrupted; it is in the same line with [42], which is likely to be a kind of cyberbullying. The chances of cyberbullying decrease. This study suggests that cyberbullying is a statistically important relationship (*P* < 0.05) between knowledge and age of studied students. Because older students are less likely to be involved in aggressive or harmful behavior directed at a person using any form of electronic communication. This may be since older students have more experience using technology and social media, whether online or offline, to conduct their assignments and are more capable of avoiding cyberbullying. There is a statistically important relationship (*P* < 0.05) between cyberbullying approaches and the academic year of students, respectively.

This is [51], which comes with a reported growing relationship between academic levels and cyber victims in high school and university adolescents. It also discovered [52], who found that the year (student’s position) in school was envisaged to have a relationship with cyberbullying, and students who have been cyberbullied or who are victims of cyberbullying in high school are more likely to become victims or bullies in college. Also [53], found that cyberbullying emerges from a complex interplay of individual, social, and technological factors. Its impact extends beyond psychological harm to victims, affecting academic performance, social development, and, in severe cases, resulting in self-harming behaviors.

Regarding cyberbullying approaches and beliefs, this study found that the students had an adverse attitude towards cyberbullying of most of the students, while only less than one quarter had a favorable attitude towards cyberbullying. It agrees with [54]. He showed that the largest percentage of the study sample was a negative attitude towards famous celebrities-caborbuling (62% of total samples) and had fewer practical intentions to reduce it (67.5% of total samples), most samples believed that it was a moral perspective (65% of total samples). Cyberbullying is a highly statistically significant relationship (*p* = 0.00) between cyberbullying dynamics and risk factors, cyberbullying approaches and beliefs and cyber-orientalism behavior.

The result in this regard agrees with [55] who found a positive relationship between cyberbullying victims and cyberbullying crime among university students. This study indicates that the students had low cyber-aggression behavior with the majority of the studied, while 12% and 6.75% had moderate and high cyber-aggression behavior, this discovery is in harmony with [56], which 66.5% of students reported to be low cyber victims, while 39.8% reported.

### Study strengths & limitations

#### Study strengths

Understanding cyberbullying in future healthcare professionals is vital for pinpointing where to intervene and support them during their education and careers. This knowledge can help develop curricula and professional guidelines. Identifying risk factors allows educators to proactively protect vulnerable students and implement preventative measures. Ultimately, such research directly informs new educational programs, counseling services, and policy changes in nursing schools and healthcare institutions, filling a crucial gap in broader cyberbullying research. However, this type of research has limitations. Using self-reported questionnaires can lead to social desirability bias (underreporting to appear resilient) and recall bias (difficulty remembering past events). A cross-sectional design only shows associations, not cause-and-effect.

#### Study limitations

Findings may lack generalizability due to varying cultural norms and policies across different nursing colleges or regions. Additionally, our study did not include direct measures of individual psychological factors, such as specific personality traits or mental health statuses, which could serve as confounding variables. While we aimed to control the study environment, these inherent individual differences may have influenced participants’ experiences and responses to cyberbullying. Future research would benefit from incorporating validated measures of these psychological constructions to provide a more nuanced understanding of their role in cyberbullying dynamics among healthcare professionals.

#### Implication to nursing education

This study highlights the urgent need to boost cyberbullying education in nursing programs. Our findings show that nursing students may not have enough knowledge, appropriate attitudes, or a full grasp of what constitutes cyber aggression. To address this, nursing curricula should add specific content on cyberbullying risk factors, covering individual, social, and technology-related vulnerabilities. This will help future nurses recognize cyberbullying signs in patients. Education also needs to challenge permissive attitudes towards online aggression, fostering empathy and responsible digital citizenship through methods like case studies and ethical discussions. Finally, nursing programs must emphasize digital professionalism to curb cyber aggressive behaviors among students themselves, reinforcing ethical online conduct and its impact on a nurse’s professional reputation. Cyberbullying education in nursing practice is vital for equipping nurses to recognize, address, and prevent the negative impacts of cyberbullying on patients, colleagues, and themselves. This education helps nurses provide holistic care, enhance patient safety, and foster a supportive professional environment.

## Conclusion

This study investigated multiple aspects of cyberbullying among university nursing students, directly addressing its five core objectives. Findings revealed that a substantial three-quarters of students possessed profound knowledge of cyberbullying dynamics and risk factors, indicating a generally well-informed student body. Correspondingly, most students held unfavorable attitudes towards cyberbullying, with only a small minority expressing favorable views, and the vast majority reported low self-aggression behaviors, suggesting limited engagement as either victims or perpetrators.

Furthermore, the research identified statistically significant relationships among the key variables. A strong correlation emerged between students’ understanding of cyberbullying dynamics and risk factors and their engagement in cyber-aggression behaviors, implying that greater knowledge is linked to a lower likelihood of aggressive online acts. Similarly, a significant relationship was found between students’ unfavorable attitudes and beliefs towards cyberbullying and their reduced involvement in cyber-aggressive behaviors.

### Recommendations

Future research should explore the root causes and lasting effects of cyberbullying on nursing students and the wider community. This could involve qualitative studies to understand personal experiences in depth or longitudinal studies to observe behavioral changes over time. Nursing colleges and other educational institutions should create structured training programs for students. These programs should focus on reporting mechanisms: teaching students how and where to report cyberbullying incidents effectively, coping strategies: providing practical skills to manage the psychological and emotional impacts of cyberbullying, for both victims and bystanders and promoting positive online behavior: encouraging digital citizenship and empathy to cultivate a supportive online environment.

## Data Availability

No datasets were generated or analysed during the current study.
